# Placebo Effects in Psychotherapy: A Framework

**DOI:** 10.3389/fpsyt.2019.00456

**Published:** 2019-06-26

**Authors:** Paul Enck, Stephan Zipfel

**Affiliations:** Psychosomatic Medicine and Psychotherapy, Department of Internal Medicine VI, University Hospital Tübingen, Tübingen, Germany

**Keywords:** placebo effects, psychotherapy, control condition, placebo response, clinical trials

## Abstract

The issue of placebo response and the extent of its effect on psychotherapy is complex for two specific reasons: i) Current standards for drug trials, e.g., true placebo interventions, double-blinding, cannot be applied to most psychotherapy techniques, and ii) some of the “nonspecific effects” in drug therapy have very specific effects in psychotherapy, such as the frequency and intensity of patient–therapist interaction. In addition, different psychotherapy approaches share many such specific effects (the “dodo bird verdict”) and lack specificity with respect to therapy outcome. Here, we discuss the placebo effect in psychotherapy under four aspects: a) nonspecific factors shared with drug therapy (context factors); b) nonspecific factors shared among all psychotherapy traditions (common factors); c) specific placebo-controlled options with different psychotherapy modalities; and d) nonspecific control options for the specific placebo effect in psychotherapy. The resulting framework proposes that the exploration and enumeration of context factors, common factors, and specific factors contributes to the placebo effects in psychotherapy.

## Historical Roots

Although the term “placebo” became commonplace medical language some time ago (1), it was not before the 1940s that placebo-controlled pharmacological trials became the standard in psychiatry and beyond (2). This rather restrictive use of the term for controlled trials was relinquished only recently in favor of a broader use in all therapeutic conditions, for differentiation between minimizing placebo effects in controlled trials, while maximizing it in daily routine (3, 4), and for harnessing the effect to improve the therapist–patient relationship (5).

Throughout this paper, we will use the terms “placebo effect” and “placebo response” (or “nocebo effect” and “nocebo response”) in accordance with a recent expert opinion of the placebo research community (4): Placebo effect refers to a distinctive psychobiological phenomenon, while placebo response refers to the outcome of clinical trials, the amalgam of responses after receiving a placebo—bias in reporting, regression to mean, possibly also Hawthorne effects, and placebo effects (6).

However, psychotherapy and the placebo response share a specific and delicate relationship.

A response to placebo was soon recognized as an indication of a psychological rather than of a somatic/medical condition (7). Two “roots” of this early placebo research can be identified:

- In the early 1950s, Stewart Wolff described the mechanisms (conditioning, expectation) by which placebo effects occurred and were strong, particularly with somatic symptoms such as pain and nausea (8, 9). At the same time, in psychiatry, particularly high placebo effects were observed in randomized controlled trials (RCT) with drugs in depression, anxiety, etc. (10), and among other things, the severity of the illness, duration of treatment, and previous therapies were causing this effect (11) [for a survey, see Weimer et al. (12)].
- Around the same time, Jerome D. Frank noted that patients’ and therapists’ expectations influenced the outcome of psychotherapy (13) and speculated that suggestions (but not motivation) may play a role, as may the duration of therapy, specific-patient characteristics (which he called placebo reactors), and side effects may eliminate it. To distinguish between specific and nonspecific effects, Frank called for clearly defined control groups in psychotherapy also, regardless of its theoretical orientation.

**Figure 1:**
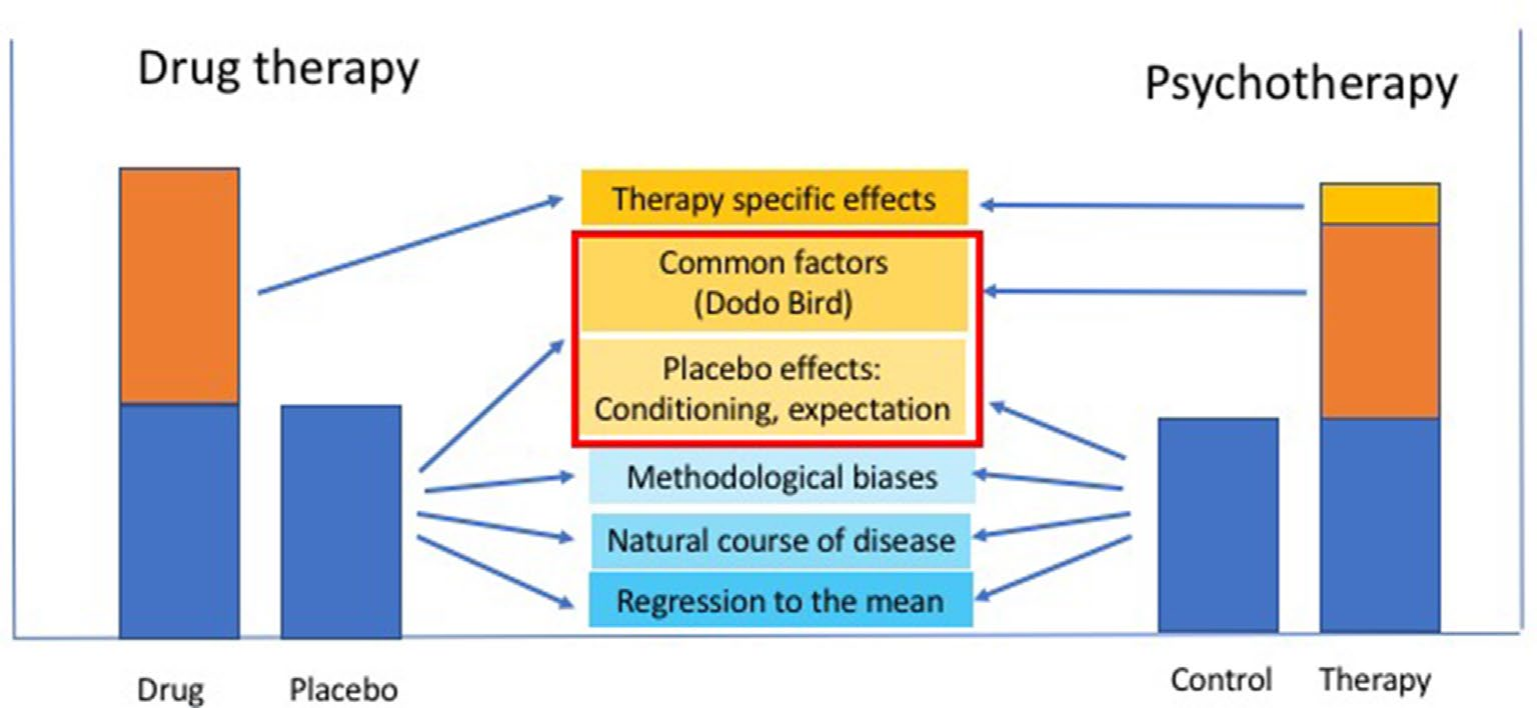
Schematic relationship between shared and non-shared nonspecific factors contributing to the placebo response in drug and psychotherapy: factors that are part of the (nonspecific) placebo effect in drug therapy (e.g., therapist empathy, intensity of patient-therapist communication, etc.) become “common factors” across all psychotherapies [Rosenzweig’s “Dodo Bird” (1936), or Lambert and Ogles’ “common factors” (2004)], addition to a (small) specific effect of the different psychotherapy modalities that may be composed of a specific combination of the factors, as listed in **Table 1**.

Little has been achieved experimentally since then with regard to exploring placebo effects in psychotherapy, although the therapeutic options available have increased dramatically: psychodynamic psychotherapy, cognitive behavioral therapy (CBT), hypnotherapy, interpersonal psychotherapy, group therapy, couple and family therapy, mindfulness-based therapy (MBT), self-help programs (SHPs), phone- and internet-based therapies, health interventions, e.g., smartphone apps, and the like. The general and specific placebo effects of all of these should be examined. In the following sections, we will argue that of the many factors regarded as “nonspecific” in drug RCT, some should be considered as being specific in psychotherapy, while others remain nonspecific under all circumstances. As with drug therapy, however, not all nonspecific factors are attributable to a placebo effect; since response biases, statistical regression to the mean and spontaneous symptom variation account for some of the effects involved in both the drug and the placebo aspect of trials and therefore also influence psychotherapy. This concept is illustrated in **Figure 1**.

While most of the older and many recent publications on the placebo effect in psychotherapy avoid determining the size of the placebo effect in psychotherapy (14–18), unless they were claiming that the placebo concept cannot be applied to psychotherapy at all (15, 19), others argue that properly designed placebo (control) therapy may be as effective as psychotherapy (16). However, neither of these positions is helpful in planning a rational psychotherapy evaluation.

Instead, we will follow an argument raised by Blease (20, 21); according to which, there is general scientific consensus that the placebo concept exists, but unnecessary debate in placebo studies persists due to the failure to recognize this fact. In principle, the same underlying definitions for placebo response and placebo effect that apply in biomedical research interventions also apply to psychological interventions for which the concept “placebo” was not developed. The key difference lies in recognizing the serious challenges of placebo-controlled clinical trials for psychological treatments. It is therefore unnecessary to eliminate placebo concepts in psychological contexts, as proposed by Kirsch (15).

We will abstain from discussing the placebo concept of Grünbaum (22) for CBT for one simple reason: it was developed before the surge of empirical placebo research had begun in the 1990s (23) and thus cannot reflect current knowledge. Gaab (19) falls into the “Grünbaum trap” when arguing that psychotherapy is at risk of being misconstrued as “mere” placebo without such a discussion and that psychotherapists otherwise simply prescribe placebos. It is not without irony that Wampold (16) illustrates the concept with a contemporary drug example (antibiotics) but falls short (as do others) of explaining what contemporary “incidental constituents” of psychotherapy may be, adhering instead to Grünbaum´s 1986 definition.

A “Grünbaum trap” is what we call the outdated understanding of the placebo response in psychotherapy. It was developed as a seemingly timeless concept (applicable to all psychotherapies at all times, e.g., the “incidental constituents of psychotherapy” according to Grünbaum) when much of what determines the placebo response had already been identified, e.g., learning history and acute expectancies, which are no longer “incidental” in either drug therapy or psychotherapy.

Our subsequent arguments assume that—like drug RCT in similar conditions, when primary efficacy measures are patient-reported outcomes (PROs)—an average placebo response of around 40% may also be effective in psychotherapy, provided that optimal research conditions prevail; where this is not the case, the placebo response is liable to be higher. This position is supported by a more recent meta-analysis of psychotherapy trials in irritable bowel syndrome (IBS) with nearly 100 RCT of drug therapy and an average of 40% placebo response across all trials (24); six additional psychotherapy RCT also yielded an average placebo response of 40% (25).

This is similar to Lambert (26) who proposed that 40% of the effect of psychotherapy is attributable to factors beyond psychotherapy (or, in our terms, nonspecific effects: spontaneous variation, regression to the mean, biases) and a further 15% to the placebo effect (expectancy of improvement); in addition, 15% are thought to be due to the specifics of each psychotherapy modality, while the remaining 30% are common to all psychotherapies, the “dodo bird verdict” (27). These 30% “common factors” of all psychotherapies can be subdivided into “support factors,” “learning factors,” and “action factors,” in accordance with Huibers and Cuijpers (28) (see **Table 1**).

**Table 1:** Factors assumed to be common in all psychotherapies that may influence psychotherapy outcome. These can be classified in three groups and can—to different degrees—be effective in different psychotherapies, thus enabling different modes of psychotherapy to operate. Their sequential order (from left to right) is based on a concept by Lambert and Ogles (29) that is theory-driven and yet without empirical basis [concept according to Huibers and Cuijpers (28)].

Interaction factors	Process factors (Learning)	Process factors (Action)
*Matching of patient/therapist:*	*Emotional dimension:*	*Behavior:*
identification	accepting advice	practice
therapeutic alliance	affective experience	taking risks
therapist´s expertise	assimilation of problems	facing fears
active participation of both	correction of emotionality	mastery efforts
structured communication	allowing emotionality	experiencing success
*Positive relationship with:*	*Cognitive dimension:*	*Behavioral regulation:*
Trust	Feedback	modeling
Empathy	Rationality	reality testing
acceptance	allowing insight	working through
reassurance	cognitive learning	cognitive mastery
genuineness	identifying expectations	predicting problems
release of tension	changing expectations	designing future solution
mitigation of isolation		

While all these numbers may be variable with respect to their empirical base—from guesses to meta-analyses—they come surprisingly close to what has been reported from RCT across medicine (30) as well as from psychiatry (12) and in the range of what Henry K. Beecher had already estimated from the few clinical trials he had at his disposal in 1955 (7). Provided that PRO are in the focus, our current understanding is that at this level, placebo effects in drug therapy and in psychotherapy do not vary whatsoever in size and mechanism.

We will not elaborate further on the concept of these “common factors”—a detailed review and discussion is available in Lambert (26). An in-depth discussion of the control-group issue in psychological interventions can be found, among others, in Mohr et al. (31) and Guidi et al. (32).

We will neither present nor discuss the vast body of evidence with regard to neurobiology and neurochemistry of the placebo response, but again refer to the literature, e.g., Fabrizio Benedetti´s book (33), and Luana Colloca´s reader (34, 35).

We will structure the following discussion using the analogy of drug therapy and aim to identify nonspecific effects in drug therapy that have either become specific or that have remained nonspecific in psychotherapy. We will discuss common problems of control for nonspecific effects across different psychotherapies as well as potentially specific problems in certain psychotherapies, as also illustrated in **Figure 1**. Finally, we will address placebo issues with a combination of drug and psychotherapy and discuss the relationship between placebo effects and the efficacy of psychotherapy.

## Nonspecific Effects in Drug Therapy Which Become Specific in Psychotherapy

Most RCT with drugs are keen to demonstrate that no center effects occurred, which otherwise could explain to some degree the efficacy of the drugs under investigation. In pivotal trials, such a center effect could potentially cause the requested indication to be declined by the approval authorities. It is of interest to note that in RCT before the 1990s, most studies were single-center trials in which such an effect was not even noticed. Furthermore, the qualification of trial doctors, the degree of their training, and their communication skills and empathy were rarely assessed or subsequently linked to treatment outcome. Age, sex, and other personal characteristics of the patients were not specifically taken into account, although it is well established that these factors may play a role in clinical routine (36) as well as in RCT, for both drug therapy (37) and psychotherapy (38). Rules of good clinical practice required independent raters and therapists, staff training, study monitoring, and strict adherence control (39).

Large multicenter trials produce higher placebo responses (40–42), presumably due to a lower standardization of recruitment [including recruitment biases (43)] and higher variability of therapist–patient interaction during the study. In agreement with this, more study visits are now clearly associated with higher placebo response rates in depression in both children (44) and adults (45), as well as in other areas of medicine, e.g., inflammatory (46) and functional bowel disorders (24).

Frequency and intensity of therapist–patient interaction are well-known factors determining the efficacy of psychotherapy (47). They may serve as an example of how nonspecific effects in drug therapy could become specific effects in psychotherapy, however common they may be for most psychotherapy modalities. This is why psychotherapy trials have always sought to standardize the amount of time spent with the patient as well as the communication between patient and therapist. Furthermore, while manuals harmonizing the content and interaction during therapy are standard in psychotherapy, such factors are now also deemed to be relevant in drug trials (48).

One prime example of a common but specific effect involved in psychotherapy is described in an open-label placebo study (49): To achieve an “augmented placebo response” in a sham-acupuncture trial in patients with IBS, acupuncturists were instructed to control their treatment behavior on the basis of a manualized script requesting intensified 20-min doctor–patient communication instead of the usual, standard acupuncture treatment. Many of the verbal instructions required “normal” therapist–patient communication behavior in a psychotherapy setting but may be rather atypical in drug therapy environments.

This procedure doubled the placebo response to sham acupuncture on most outcome measures.

## Nonspecific Elements in Drug Therapy That Remain Nonspecific in Psychotherapy

Of the small number of patient-centered predictors of the placebo response identified in RCT in psychiatry (12)—low severity of the disease, short disease duration, no treatment history, more recent trials—none were shown to be specifically relevant in psychotherapy, although it is open to speculation as to whether patients accepting psychotherapy as their primary treatment option are less severely affected, e.g., by depression, than patients accepting psychotropic drug therapy. In depression therapy in particular, younger age was associated with higher placebo response, but this may be due to shorter disease history and lower disease severity in children and adolescents than in adults (30, 50). These factors may lose their importance in all those cases in which a first-line drug therapy is not available.

Of the traditional therapist-centered variables tested (age, sex, theoretical orientation, and percentage of work time conducting therapy), only age was a significant demographic predictor of psychotherapy outcome in a univariate analysis, while in a multivariate analysis, interpersonal and social skills accounted for most of the outcome variance (51). While this casts doubts on the replicability of many psychotherapy RCT, it calls for more research into the role of researcher variables for therapy outcome (52): allegiance to theoretical concepts per se has been made responsible for most of the therapy outcomes (53).

It is, however, of relevance that, particularly in psychiatry—but not outside psychiatry, see Ref. (54)—an unbalanced randomization has been shown to drive the placebo effect: increased placebo effects were observed in depression (45, 55, 56), schizophrenia (57, 58), and psychosis (42) when more patients were randomized to active treatment than to (placebo) control. While this is usually carried out for ethical reasons (to leave the least number of patients untreated), it also serves in certain cases to test different drug dosages against one placebo arm.

Such designs are presumably also common in psychotherapy and may account for a substantial overall effect of the therapy: According to Papakostas and and Fava (55), a 10% increase in the probability of receiving active treatment (i.e., a 10% decrease in the probability of being assigned to the control condition) increases the probability of responding to active (drug) antidepressant therapy by 1.8% and to control (placebo) by 2.6%, in comparison with a 50:50 randomization scheme. When one active treatment is compared with another active treatment [comparative effectiveness research (CER)], the response was higher by a factor of 1.79 than in a placebo-controlled trial, solely brought about by the 100% certainty for patients that they would receive active antidepressant treatment (59).

## Common Control Problems in All Psychotherapy Trials and Their Advantages and Pitfalls

Different psychotherapy options share common features when it comes to standards as set down by RCT of drug therapy in psychiatry and psychosomatics, e.g., trial registration, power calculation, ethics approval, and informed consent are easily applicable to all. Others, such as monitoring of treatment progress and adherence control need to be adapted to the specific therapy in some cases, e.g., with internet-based therapies. In most cases, the design also required adaptation to specifics for certain therapy options (60).

The common denominator in all psychotherapy procedures is the inability to effectively blind treatment and control group assignment and to provide a “true” (by nature, ineffective) placebo treatment; among the many procedures that have been developed to secure blinding therapy assignment and to warrant equipoise (61), very few are applicable to psychotherapy (62). Both limitations have important consequences for the placebo response, as will be discussed later. Nevertheless, current guidelines for good clinical practice require independence of raters and diagnostic staff and their impartiality toward the intervention (39).

### Ineffective Blinding

Blinding (of the patient) as well as double blinding (of both patient and therapist) is literally impossible, not only in psychotherapy but also with many other interventions such as manual or physical therapy. Even where apparently possible, e.g., in biofeedback and neurofeedback therapy where “false feedback” (signals from another patient, e.g., as “yoked control”) is provided, patients will realize immediately whether they have been randomized to treatment or control. The situation mimics some of the circumstances encountered in therapies using technical tools, e.g., acupuncture, transcutaneous electric nerve stimulation, and transcranial magnetic stimulation where only those patients can be enrolled who had never experienced the “real” therapy before and who may possibly be hoodwinked (63).

In classical drug RCT, unblinding will have imminent consequences for efficacy. Deliberate unblinding of RTC is usually only carried out when severe safety concerns arise but may also occur incidentally when patients and/or doctors notice significant differences in reporting of adverse events (64); even meta-analyses can identify such involuntary unblinding (65). Such unblinding will enhance the response to active therapy and reduce the response to control, thus enlarging the treatment–control difference (66). However, when therapies with either double-blinded placebo-controlled drug interventions or unblinded but controlled psychotherapies for the same condition (depression) were compared, the meta-analysis showed a small but significant effect (drug–placebo difference) in favor of pharmacotherapy (67), indicating that (un-) blinding affects psychotherapy to a lesser degree than conventional drug RCT. Furthermore, patients who were obviously assigned to the control condition (irrespective of its form) will respond with disappointment (68), increased risk of dropping out (69, and potentially with nocebo effects (70), further contributing (via the “last value carried forward” requirement for intent-to-treat analysis of the trial data) to an overestimation of the efficacy of the active arm of the trial.

Blinding is particularly necessary with conventional crossover designs where each patient serves as his/her own control, thereby reducing the data variance and making RCT possible with considerably less patients than with a parallel-group design. However, crossover designs carry another risk: that of carry-over effects from one phase to the next. If the carry-over effect is based on the Pavlovian conditioning of responses (71), even the use of longer washout phases cannot prevent it from occurring.

Ineffective blinding and carry-over effects that cannot be washed out therefore constitute the two reasons why psychotherapeutic trials cannot employ a crossover design. The limitations of a parallel-group design, in particular higher between-subject data variance, had to be overcome by developing other design features to account for the missing “true placebo” in psychotherapy, predominantly “waiting list control” (WLC), and “treatment as usual” (TAU).

### Waiting List Control

The fact that no “true placebo” is applicable in psychotherapy RCT does not imply that no placebo effect occurs, as discussed above in the case of CER: when psychotherapy is compared with another therapy, the placebo effect is not controlled for and can therefore no longer be quantified. It can, however, be assumed that some of the alternative control strategies in psychotherapy research also have enhancing placebo effects. This may specifically be true of WLC.

Like crossover studies, WLC reduces data variance on account of a lower within-subject than between-subject variability of data; in this case, however, all patients should have to wait (which is usually not the case). It is also argued that WLC may additionally serve as a control for spontaneous variation of symptoms, a condition that cannot be readily tested with any RCT: it is ethically questionable as to whether a “no treatment control” is acceptable unless the disease is of minor severity and no effective therapy is available. This is the most rigorous interpretation of the current position of the Declaration of Helsinki (72).

Three-arm trials (active, placebo, and no treatment), between 25% and 45% of the treatment effect—of either drug or psychotherapy—can be attributed to spontaneous variation (73), with highest effects in nausea (45%), smoking cessation (40%), depression (35%), phobia (34%), and acute pain (25%). The authors concluded that most of the placebo effects in these conditions are attributable to spontaneous variation of symptoms. However, Kirsch and Sapirstein concluded in their initial paper (74) that 25% of the improvement observed in the drug-treated group (for depression) was due to the active medication, 25% to natural history, and 50% to the placebo effect.

The use of WLC as an indicator of spontaneous variation is therefore misleading (patients are not naive but are promised effective treatment), and it would be more appropriate to install one of the novel designs that separate recruitment for a disease-monitoring study from recruitment for an intervention study, called Zelen design (75) or multiple cohort RCT (MCRCT) design (76) (for more details, see 77 and 78).

Since WLC are promising patient-effective treatment in the future, they may produce strong expectancy effects, probably enhancing the placebo response, even in the phase before the treatment actually commences: symptom improvement during waiting has been reported (79, 80)—similar to effects of run-in periods in drug trials (81)—and cannot be taken solely as indicative of spontaneous remission. Placebo-controlled trials are superior to WLC trials and induce greater symptom reduction (82), as do RCTS with a “no treatment” condition in comparison with WLC trials (70). Furthermore, this effect may rely on the duration of waiting, and standard rules for this have yet to be investigated. One way of doing so would be to install a “step-wedge approach” (83), where randomization between different waiting groups (periods of different length) is used to test a dose-response function of waiting and the point at which positive expectations (placebo effects) may turn into disappointment (nocebo effects) (70) and increased dropout rates (69).

### Treatment as Usual

If being randomized to a WLC can induce hope (placebo) or disappointment (nocebo) depending on its length, being randomized to TAU, by dint of its name, is already suggestive of its nocebo effect, the implicit message being that “you get what everybody else gets with this disease, and this treatment is unsatisfactory; that is why we are testing the new one, to which you, unfortunately, have not been randomized.” Unless this is a treatment-naive patient with a very short disease history [which is indicative of high placebo response rates in many conditions and with many therapies (12)], this also reminds him or her of previous unsatisfactory or unsuccessful therapies, which—as we know—contributes significantly to the efficacy of any novel therapies (84).

Being randomized to the active treatment arm rather than to TAU will therefore enhance the placebo effect by enabling patients to compare the ongoing therapy with (all) previously inefficient therapies. For this reason, they prefer to participate in the novel approach; being randomized to TAU is almost a verdict. A TAU approach therefore enhances the placebo effect in the active arm and induces nocebo effects in the control arm.

The only thing that we have learned for sure from placebo/nocebo research over the past 10 years is that words can be painful (85) and can induce nocebo effects (86, 87). TAU definitively hurts, and it would be better rephrased as “the best available and approved treatment” (BAAT) when compared with a novel approach, but this would not work without having a number of logistic repercussions for trial designs.

Firstly, the utmost standardization of the TAU/BAAT treatment used for control purposes would be required. However, this is usually not carried out in psychotherapy RCT involving TAU. It is particularly complicated when patients are recruited from different clinical settings for treatment in a specialized center, but TAU is provided by the transferring therapist. It also generates a further methodological issue with regard to the selection of BAAT, when more than one is available on the market, in the region, or under prevailing restrictions, e.g., health insurance plans. It should be noted that control conditions, e.g., optimized treatment as usual (TAU-O) in psychotherapy trials [see for instance Refs. (88, 89)], face additional challenges, depending on the health care environment in which they were conducted. In Germany, by way of example, patients with a psychiatric or psychosomatic illness, e.g., anorexia nervosa, have access to inpatient, day-patient, and outpatient psychotherapy treatment. If these patients are randomized to the TAU-O arm, they have a choice with regard to a) the treatment setting, b) the treatment method (e.g., CBT or psychodynamic psychotherapy), c) the therapist, and d) the intensity or dosage of therapy. It is therefore also particularly important to discuss findings of studies on the background of the health care system in which they were conducted. These challenges are somewhat similar in CER, where divergent interests (drug companies, ethics boards, and patient representatives) may nominate different options as BAAT (90). Given the large number of different psychotherapeutic approaches to one disease, this may be impossible to achieve in RCT but perhaps in meta-analyses of RCT (91). Finally, if one novel treatment A is compared with the best (or one of the bests) treatment B, statistics cannot rely on A’s superiority over B but should test A’s non-inferiority in comparison with B, with the consequence that as many as a fourfold number of patients could be required to confirm this (92). Not to mention the fact that this generates an ethical paradox since, according to the Declaration of Helsinki, the smallest possible number of patients should be recruited for a trial, while all others should receive regular and adequate treatment (93).

Among the many design alternatives that have been developed to either explore and maximize the placebo response or to avoid or minimize it in drug RCT (3), the so-called preference design may indicate an alternative approach specifically relevant for psychotherapy (78). In short, patients can choose between two (or more) alternative therapies, e.g., drug or psychotherapy, and are assigned accordingly (94). Only those who have no clear preference will undergo randomization. The role of patients’ preference (and its placebo effect) can be assessed *post hoc*, comparing those with a preference for the one therapy with those randomized to this therapy in each therapy arm.

### Specific Control Problems with Specific Psychotherapy Modalities

Beyond these general problems of control conditions with global placebo and nocebo effects in psychotherapy RCT, specific psychotherapies generate specific problems related to control and adequate estimation of the placebo/nocebo effects. Much of what has recently been described as decisional framework for neurocognitive and behavioral intervention (60) applies to most other therapeutic options also.

The subsequent review of different psychotherapies that we discuss bears some arbitrary selection bias and may reflect a more traditional vision of the spectrum of psychotherapies available. However, the intention is to illustrate rather than to cover the variability of problems associated with specific psychotherapy modalities. Readers who feel neglected or overlooked are welcome to consider and outline the specifics of their own modus operandi in light of what has been discussed.

### Psychodynamic Psychotherapy

As already observed by Frank (13), it is essential for any control strategy attempting to catch the placebo effect in psychotherapy RCT that it devotes the same interaction (time, number of contacts, and intensity of communication) between the patient and the therapist as is the case in the “active arm” of the therapy. He proposed the use of relaxation therapy as a control for psychodynamic therapy (PDT), but it may equally well be any other passive but interactive therapy. It should be borne in mind that, in most such cases, the control condition does not provide a clear measure of the placebo effect but simply another effective therapy. This will increase the pressure to demonstrate superiority of PDT over control while increasing the placebo effect in both arms. Recent approaches (52, 88) applied standardized diagnostic systems (e.g., operationalized psychodynamic diagnosis) to identify clear treatment foci on the basis of a psychodynamic approach (52, 88).

### Cognitive Behavioral Therapy

Unlike psychodynamic psychotherapy (PDT), CBT approaches intrapersonal problems at an individualized rational (cognitive) or behavioral level on the basis of an extensive prior behavioral analysis. To adequately control for nonspecific effects, it is feasible that written information on putative cognitive and behavioral strategies that are independent of the patient’s own history may provide a control strategy. Albeit this lacks the actual behavioral analysis that precedes the active part of the therapy, it is, nevertheless, part of it. Behavioral exercises and tasks, which may mimic some of the effects occurring, bear the risk of errors if not adequately structured to the patient´s pathology and therefore require careful monitoring.

### Interpersonal Psychotherapy

Interpersonal psychotherapy, like CBT, is based on a very intimate knowledge and guidance of the patient’s acute problems, and problem solving may therefore not tolerate “sham” interventions without becoming evident. Keeping an (electronic) diary may be a method of monitoring one’s own problems in the absence of a therapist (95). MBT, self-aid programs, and educational programs may also provide a lower-level control for the attention received.

### Mindfulness-Based Therapy

An increasing number of therapy studies have demonstrated the efficacy of MBT in somatoform disorders such as IBS. Meditation-based therapy is difficult to control for nonspecific effects. In some trials, validated self-aid programs are used for attention control (96) or “sham mindfulness meditation” (97).

### Couple and Family Therapy

The nonspecific contribution of “proxies” toward therapy efficacy has been well established for children in medical therapy (98) but has rarely been assessed in adults (99). Experimentally, the placebo and the nocebo effects in both groups are affected by social models, be it peers, parents, or strangers (100, 101). The control strategies therefore become even more difficult when the “proxies” are part of the intervention, as is the case in pair and family therapy. Merrilees et al. (95) used event-contingent diaries about marital conflict situations to change marital interactions as a control strategy rather than conventional face-to-face family psychotherapy sessions.

### Group Psychotherapy

The problem of “proxies” and “others” for the therapy progress and success of individual patients becomes even more virulent with group psychotherapy. One control strategy would be to run two (or more) groups in parallel, with all participants truly randomized to one of the groups, and to compare the group as well as the individual progress between the two. In addition, group processes could be monitored by applying group-specific outcome measures. A further control strategy could consist of using eHealth applications such as chatrooms, focus groups, self-aid guides, and blogs as controls (102).

### Hypnotherapy

Nonspecific effects of hypnotherapy, whether general or in a disease-specific form such as gut-directed hypnotherapy (103), are probably best and most readily controlled by relaxation exercises and therapy, since these are similar with respect to the time spent (in a group setting as well as in individual therapy) and active/passive components. A comparison with mindfulness mediation (see previously), while perhaps advisable, has not yet been conducted.

### Self-Help Programs

SHPs were initially developed as a control condition for more manualized therapies, especially in patients with somatoform disorders, such as the IBS (104). As they developed their own theoretical framework, and for economic reasons—providing professional help to more patients outside academic centers—many applications are now available, particularly in combination with web-based approaches (105).

### E-Mental Health Approaches

The very recent development of phone- and internet-based therapies has spread across all psychotherapy modalities, from CBT to MBT and SHP, e.g., Refs. (106) and (107). Among the most widely used applications is Deprexis^®^, an internet-based CBT program for the treatment of depression (108, 109). Due to its high standardization, it can easily allow for the control of the effect of a variety of nonspecific factors such as age, sex/gender, race, and other therapist-based demographics, for style of communication (personalized versus neutral), for intensity of communication, e.g., with or without question and answer, feedback, chatroom activity, etc. By contrast, of the many smartphone health applications presently available (now numbering over 300,000), those with a psychotherapeutic approach still lack clear control strategies that would enable us to estimate the overall efficacy of their placebo effect (110). Using “virtual” doctors or therapists (111) in the future may enable us to exert a much better control of the nonspecific factors not only in psychotherapy but also in medical therapy in general (21, 112).

## Nonspecific Solutions for the Control of the Specific Placebo Effect in Psychotherapy

In a recent paper (60), we proposed a dynamic decision framework for choosing a control condition depending on the patient population and associated risks, i.e., the risk of the disease itself, placebo vs. nocebo responses in this population, and the armamentarium of available therapies with known efficacy for this patient group, as well as the trial stage. We argued that the choice of control group and its justification need to be taken into consideration, e.g., when comparing behavioral and pharmacological therapies. High participation risk studies should therefore choose among controls with high effect sizes favoring treatment (e.g., waiting list and TAU) that may require smaller sample sizes, while low-risk studies may opt for active comparators and minimal treatment control conditions [see Figures 1 and 2 in Ref. (60)].

Wampold characterizes three global strategies resulting from the need to control for nonspecific effects of psychotherapy: a) identifying single components of the psychotherapy under investigation and replacing them by components of another psychotherapy (tradition); b) dismantling, without replacing, one or more components of a specific psychotherapy; and c) using treatments that control for common factors such as education and counseling (16).

Neither strategy has produced convincing results when it comes to adequately controlling the placebo effect in psychotherapy and has (worst case) shown that the control therapies may be as effective as the therapy under investigation, e.g., Ref. (113). Another novel control strategy, known as “befriending” (114, 115), refers to professional (nurse-conducted) social contacts developed for patients with schizophrenia in the community (116). It may, however, fall into the same trap as others before it, in demonstrating that even the mildest form of patient–therapist communication can result in significant therapeutic effects (117) and may therefore be a specific control only for the specific group of patients for which it was developed.

The “Goldilocks placebo effect” (118) exploits something that has rarely been tested and compared in psychotherapy research, i.e., the provision of alternatives from which the patient may choose. Preference designs (94) allow patients to choose between alternative treatments when available (e.g., drug vs. psychotherapy, different psychotherapy options) prior to randomization. It also allows comparison of the efficacy in patient that preferred one treatment arm with patients that were randomized to this arm of the study. The role of preferences can also be included in the overall statistics when comparing both treatment effects (78). Although the use of preferences does not seem to affect the overall internal and external validity of trials (94) and the preferences themselves do not appear to play a role in the placebo response (119), the systematic evaluation of placebo data beyond acupuncture has not yet been carried out.

By way of comparison (antidepressant versus CBT) of treatment outcome (treatment–control difference, not of the placebo response) in patients with depression, patients in either arm who selected this treatment were found to respond better than those who were randomized to the same arm (120). The difference was even greater in CBT trials and was independent of depression severity and dropout rates. In a trial in patients with chronic widespread pain, participants could choose between four options (CBT, exercise, a combination of both, or TAU), and the treatment preference had no effect on treatment outcome, while improvement expectations did (121). Neither of the studies elaborated on the placebo effect size under preference–choice conditions.

The “Goldilocks principle,” which refers to Goldilocks´ quote about her preferred porridge temperature as being “just right” in the popular fairytale “Goldilocks and the Three Bears” (Robert Southey 1837), has found many applications in science. The Goldilocks placebo effect study (118) takes things a step further and asks whether it is the number of options available rather than the option to select per se that determines the placebo effect, and that the effect is larger when this number is “just right” than when there are too many or too few options to choose from. While their example is taken from a choice between different alternative medicine remedy treatments (2, 12, or 38 Bach flower essences, where the middle option received the highest rating as well as the highest symptom improvement report), the principle may also apply to lower number of choice options. Whether it can explain the divergent results of the two preference studies cited previously (120, 121) remains open at this point, and more data are required before this principle can be applied to placebo effects in psychotherapy.

## Some Specifics of Psychotherapy and Its Evaluation

Unlike drug therapy, where drug–drug interactions appear to be a pharmaceutical problem only, and the placebo effects above (or below) both are identical, psychotherapy is often combined with drug therapy, albeit their interactions have rarely been investigated. An RCT with a combination of both cannot, therefore, easily answer the question as to the size of the placebo effect and the relative contribution of either component to it. In depression, for example, psychotherapy in combination with drug therapy (122) and vice versa (123) is more effective than psychotherapy or drug therapy alone. The question, however, remains unanswered. Direct comparison of drug and psychotherapy has shown no superiority of drug over psychotherapy (124), and assuming similar effect sizes of the placebo component under both conditions would not change this relationship. However, they cannot be taken as merely additive, and the superiority of the combination may be also a function of the individual patient’s preference (120). Unless an RCT is conducted and evaluated that provides either drug therapy (or placebo) alone, or psychotherapy (or an appropriate control) alone, or both in any combination, we will presumably not be able to answer this question with sufficient accuracy. Such a study resembles similarity with a double-dummy design (125), and it can be combined with a “no treatment” control group, e.g., in a register trial (76). The same holds true of other combinations of psychotherapy, such as those with neuro-modulatory therapies and biofeedback approaches (126).

Finally, one issue that requires specific consideration is the fact that, in psychotherapy, the outcome measure is usually, if not invariably a measure of subjective PRO or expert-rated outcome, whereas in drug therapy, efficacy can often be measured with both PRO/expert-rated outcome and with biomarkers, or at least with the circulating or tissue-specific level of the applied drug. Since PRO are more susceptible to placebo response than biomarkers (127), approval authorities and expert boards usually require both as endpoints in RCTs. It would therefore be advantageous if psychotherapy research were able to develop the equivalent of a biomarker as an indicator of therapy success and as an adjunct measure of the size of the placebo response in psychotherapy in the future.

## Summary

Overestimation of the efficacy of interventions, not only in psychotherapy, is common to all medical subspecialties, as is the effort to minimize nonspecific treatment effects, among which the placebo effect has the poorest reputation. Unfortunately, as we have already shown previously, psychotherapy lacks a true placebo intervention, and some of the nonspecific effects in drug therapy, such as the empathy of the therapist and the quality of the patient–therapist communication, become quite specific effects in psychotherapy. On the other hand, many of the common control strategies in psychotherapy research, especially waiting list and TAU, tend to inflate these nonspecific effects at the expense of already reduced overall efficacy, be it specific for individual psychotherapy modalities or for a “common effect” of all of them.

Under these circumstances, the scientific community of placebo researcher should not seek exemption from the scientific rules of treatment evaluation that have been developed for drug therapy but rather seek specific strategies to control at least some of the elements of psychotherapy that are responsible for the placebo response. These strategies can either be specific to certain psychotherapy modalities (as we have discussed) or further develop common strategies for all, bearing the possibility of covering at least some of Grünbaum´s “incidental constituents” without attempting to identify and enumerate them all. For instance, changing the waiting-list control into a “step-wedge” design, evaluating the cohort multiple RCT design, or developing the preference design to a Goldilocks approach for psychotherapy are empirical ways of proceeding (78, 114, 115) and much more appropriate than reasoning why it is impossible to control placebo effect in psychotherapy or remonstrating that all of psychotherapy is only placebo.

Last but not least: Biomedicine has learned to accept that placebo/nocebo effects exist outside placebo-controlled trials in daily medicine, and they contribute to a large extent to the success or failure of patient treatment, sometimes even more so than the drugs available. It is now time for psychotherapists to accept them in their daily practice.

## Author Contributions

PE had the idea for the paper and conceptualized it. PE and SZ wrote the paper.

## Funding

Supported by the German-Norwegian Günther Jantschek Research Stipend (PE).

## Conflict of Interest Statement

The authors declare that the research was conducted in the absence of any commercial or financial relationships that could be construed as a potential conflict of interest.
